# Sonication protocols and their contributions to the microbiological diagnosis of implant-associated infections: a review of the current scenario

**DOI:** 10.3389/fcimb.2024.1398461

**Published:** 2024-05-13

**Authors:** Natally Dos Santos Silva, Beatriz Souza Toscano De Melo, Alessandra Oliva, Paulo Sérgio Ramos de Araújo

**Affiliations:** ^1^ Departamento de Medicina Tropical - Universidade Federal de Pernambuco – UFPE, Recife, Brazil; ^2^ Departamento de Microbiologia - Instituto Aggeu Magalhães – Fiocruz, Recife, Brazil; ^3^ Dipartimento di Sanità Pubblica e Malattie Infettive - Sapienza University of Rome, Rome, Italy

**Keywords:** infections, review, sonication, diagnostic, microbiology

## Abstract

Addressing the existing problem in the microbiological diagnosis of infections associated with implants and the current debate about the real power of precision of sonicated fluid culture (SFC), the objective of this review is to describe the methodology and analyze and compare the results obtained in current studies on the subject. Furthermore, the present study also discusses and suggests the best parameters for performing sonication. A search was carried out for recent studies in the literature (2019-2023) that addressed this research topic. As a result, different sonication protocols were adopted in the studies analyzed, as expected, and consequently, there was significant variability between the results obtained regarding the sensitivity and specificity of the technique in relation to the traditional culture method (periprosthetic tissue culture – PTC). Coagulase-negative *Staphylococcus* (CoNS) and *Staphylococcus aureus* were identified as the main etiological agents by SFC and PTC, with SFC being important for the identification of pathogens of low virulence that are difficult to detect. Compared to chemical biofilm displacement methods, EDTA and DTT, SFC also produced variable results. In this context, this review provided an overview of the most current scenarios on the topic and theoretical support to improve sonication performance, especially with regard to sensitivity and specificity, by scoring the best parameters from various aspects, including sample collection, storage conditions, cultivation methods, microorganism identification techniques (both phenotypic and molecular) and the cutoff point for colony forming unit (CFU) counts. This study demonstrated the need for standardization of the technique and provided a theoretical basis for a sonication protocol that aims to achieve the highest levels of sensitivity and specificity for the reliable microbiological diagnosis of infections associated with implants and prosthetic devices, such as prosthetic joint infections (PJIs). However, practical application and additional complementary studies are still needed.

## 1 Introduction

Joint replacement surgeries, known as arthroplasties, are increasingly frequent and widely used procedures with the aim of replacing, remodeling or realigning a joint (Torres et al., 2015; Filho et al., 2020). Taking into account projections on certain orthopedic procedures, for example, by 2030 in the United States, a significant increase in the number of primary hip (174%) and knee (673%) arthroplasties is expected; for the same period, the United Kingdom expects a 400% increase in demand for arthroplasty (Torres et al., 2015; Ahmed and Haddad, 2019; Filho et al., 2020).

This increasing use of implantable technology has also increased the risk of deep surgical site infections (SSIs) (Torres et al., 2015). In this context, prosthetic joint infections (PJIs) occur in the joint area up to two years after surgery and are generally acquired during the implant procedure (Filho et al., 2020). They are classified according to the time interval between surgery and the onset of symptoms, which can be classified as follows: early, if it occurs within a time interval of < 3 months after the placement of the prosthesis; early late, if it occurs within a time interval of 3 to 12 months; and chronic delay, if it occurs within a time interval of >12 months. This classification also involves the way the disease is presented, whether it is acute or chronic (Beam and Osmon, 2018; Zardi and Franceschi, 2020).

Among the most common pathogens associated with PJI are *Staphylococcus* coagulase-negative and *Staphylococcus aureus*, two of which are the most common etiological agents of the disease, followed by *Streptococcus* sp., *Enterococcus* sp., gram-negative bacilli, anaerobes and yeasts. These agents are also known as good biofilm formers and are bacterial structures that are favored in PJI because of the abiotic surface of the implant and the lack of a local immunological response, resulting in persistent and progressive infection during treatment (Karbysheva et al., 2020; Zardi and Franceschi, 2020).

PJI is still considered the second most common complication, second only to aseptic loosening, and is the most important complication in arthroplasty. It may be responsible for loosening, chronic pain and instability of the prosthesis and is thus associated with a high rate of morbidity, in addition to the risk of death and the need for complex treatment strategies that involve surgical interventions and prolonged antibiotic therapy (Flurin et al., 2021; Zhang et al., 2021b). The long-term impacts on patients’ quality of life are negative; even following successful clearance of the infection, failure to control the disease can lead to the need for joint fusion and even amputation (Xu et al., 2023).

In addition to causing serious problems for the physical and mental health of patients, PJI also causes relevant economic problems. Hospital fees are generally significantly greater for the treatment of infected joints than for the treatment of noninfected joints. In the United States, the average total cost for revision knee arthroplasty is estimated at US$75,028.07, without considering the costs of prolonged antibiotic therapy at home. Similar patterns have been reported in other developed countries (Zardi and Franceschi, 2020; Xu et al., 2023).

According to statistics from the National Healthcare Safety Network (NHSN), which was released in 2017, joint infections are responsible for 1.9% of all surgical site infections (SSIs) worldwide (Moore et al., 2015). However, despite the widespread use of well-established infection prevention measures, these data on the occurrence of PJI may be underestimated due to one of the greatest challenges of this infection: diagnosis. Since there is no single test or finding for safe and accurate diagnosis, a combination of clinical findings, laboratory results of peripheral blood and synovial fluid, histological evaluations, imaging and molecular studies is performed, in addition to the important and necessary microbiological findings. In this scenario, several standardized diagnostic criteria for PJI have been proposed by different groups and societies, such as the Musculoskeletal Infection Society (MSIS), the Infectious Diseases Society of America (IDSA), the International Consensus Meeting (ICM), and the European Bone and Joint Infection Society (EBJIS), each of which adopts different definitions and cutoff points for the same infection (Trebse and Roskar, 2021).

In the process of diagnosing PJI, periprosthetic tissue culture (PTC) is considered the gold standard diagnostic technique because it allows the identification of infectious pathogen(s) and the determination of antimicrobial susceptibility, and this method can be used to determine the best and most targeted therapeutic approach (Tande and Patel, 2014; Salar et al., 2021). However, the sensitivity of tissue cultures varies from 65 to 94% and presents high false-negative rates, possibly due to the biofilm formation characteristic of this infection, which makes it difficult to obtain viable loose bacteria (planktonic) for cultivation, especially in chronic and low-grade infections preventing an accurate diagnosis from being made, causing treatment failures and prolonging the patient’s suffering (Moore et al., 2015; Shen et al., 2015).

Therefore, Trampuz et al. (2007) (Trampuz et al., 2007) popularized the use of the sonication technique to process removed knee and hip prostheses (Trampuz et al., 2007; Shen et al., 2015). Since then, sonication has been suggested as a useful method for sample processing, aiming to physically displace biofilms prior to standard culture. Organizations such as the Swiss Orthopedics and Swiss Society for Infectious Diseases (SOSSID) and EBJIS have supported its use based on studies that reported greater sensitivity and specificity of sonicated fluid culture (SFC) from explanted prostheses compared to standard culture (Shen et al., 2015; Bellova et al., 2019).

However, despite most studies in the literature indicating superior results with sonication, several studies observed a variable effect on the physical displacement of the biofilm (Bellova et al., 2019), and some even showed greater sensitivity of PTC (Oliva et al., 2016). Consequently, these discrepancies raise doubts about the reliability of the sonication technique for more accurate diagnosis of PJI (Oliva et al., 2016; Bellova et al., 2019). These variations can be attributed to the different protocols used for sonication (Oliva et al., 2016; Bellova et al., 2019).

Therefore, this report proposes an analysis of the literature on the subject in a similar way to other recent studies that reviewed the diagnostic methods available for infections associated with implants and their advances, including an overview of sonication (Birlutiu et al., 2017; Portillo and Sancho, 2023; Yilmaz et al., 2023; Azad and Patel, 2024). However, this review sought to analyze and describe the sonication protocols used in studies published in the last five years, with emphasis on the sensitivity and specificity rates achieved by these methods in comparison with PTC. Furthermore, this review also aimed to identify, in depth, the best parameters that should be considered for potential standardization of sonication protocols based on the most recent published studies.

### 1.1 Literature search

For the literature search, the following terms were used: “prosthetic joint infection,” “sonication,” “tissue culture,” “biofilm,” “sensitivity,” “specificity,” “diagnosis” and combinations of these terms. The search was conducted in the National Center for Biotechnology Information (NCBI) search engine, PubMed^®^. To comprehensively examine recent literature, the inclusion criteria for this analysis were original articles that were available electronically, published within the last five years (2019-2023) and written in English. Exclusion criteria included research such as case reports, letters, editorials and books. Furthermore, studies that addressed the microbiological diagnosis of infections other than PJI were excluded.

## 2 Sonication method

The sonication technique is performed using a device called a sonicator. This device emits sound waves in the ultrasound spectrum, creating high-intensity pressure waves in a liquid medium and causing the formation and collapse of tiny bubbles. When these bubbles collapse, they release energy capable of disrupting intercellular connections on the device’s surface, dislodging the bacteria. Additionally, sonication causes the deagglomeration and lysis of cell adhesion proteins, disrupting the physical structure of the biofilm (Oliva et al., 2016).

Due to these characteristics, sonication has been increasingly utilized to increase the yield of bacterial cultures by releasing organisms embedded in biofilms associated with implants and prostheses, particularly in joints. The sonication technique, apart from dislodging bacteria from the biofilm structure, can also lead to the lysis of bacterial cells. However, this outcome depends on various protocol factors, such as the acoustic frequency, energy, temperature, duration of exposure to ultrasound, and shape of the bacteria (Oliva et al., 2021).

### 2.1 Review/search results

We identified a total of 11 studies that met the established inclusion criteria, and these studies are described in **Table 1**.

**Table 1:** Details of selected studies.

Author / year	Type of study	Aim of the study	Methodology	Comparator	Main results
(Ueda et al., 2019)	Prospective study.	Assess whether combining the conventional culture and implant sonicate fluid culture (SFC) methods increased the diagnostic accuracy of orthopedic implant-associated infection (OIAI).	Consecutive patients (n = 66) undergoing implant removal (OIAI, 17; non-OIAI, 49) were evaluated. The total of 493 samples were analysed (39 preoperative joint aspirates, 243 peri-implant tissue specimens, 124 implant sonication, 67 controls, and 20 water bath samples). OIAI was preoperatively evaluated based on clinical evidence of infection or aspirate culture (AC). Conventional methods required positive results in either preoperative ACs or intraoperative tissue cultures (TC), whereas the combination method required at least 1 positive culture among 3 sources (AC, TC, or SFC). The application of SFC and the detection rate, sensitivity, and specificity of the diagnostic methods were assessed.	Conventional culture (Aspirate culture and Tissue cultures).	SFC alone detected OIAI in three patients (18%), with *Peptostreptococcus* and *Corynebacterium* species also exclusively isolated by SFC. The attributable detection rate of CFS infection was significantly higher than that of TC (61% vs 36%; P = 0.02). Sensitivities for AC, TC, and SFC with a cutoff of 1 colony forming unit/plaque and 1 positive culture were 60%, 59%, and 71%, respectively. When using a cutoff point of 2 positive cultures, the combined method (vs conventional) demonstrated significantly higher sensitivity (71% vs 47%; P = 0.008).
(Bellova et al., 2019)	Retrospectively study.	Determine the diagnostic performance (specificity, sensitivity) of SFC against PTC, when using European Bone and Joint Infection Society (EBJIS) criteria.	From March 2017 to April 2018, 257 implants were submitted for sonication. PJI was defined according to the EBJIS criteria as well as according to the International Consensus Meeting criteria of 2018 (ICM 2018). Only cases with at least one corresponding tissue sample were included. Samples were cultured using traditional microbiological plating techniques.	Periprosthetic tissue culture (PTC).	When using the EBJIS criteria, the sensitivity of SFC and PTC was 69.0 and 62.8%, respectively (p = .04). Meanwhile, the specificity was 90.2 and 92.9%, respectively (p = .65). When adopting ICM 2018 criteria, the sensitivity of SFC and PTC was 87.5 and 84.4% (p = .63) respectively, while the specificity was 85.1 and 92.5% (p = .05), respectively. The most commonly identified pathogens were coagulase-negative *Staphylococci* (26% overall).
(Akgün et al., 2020)	Retrospectively study.	Investigate the validity of implant sonication fluid cultures in the diagnosis of shoulder PJI compared with tissue culture.	Analyzing all patients who underwent a revision surgery for any kind of suspected septic or aseptic event due to failed shoulder arthroplasty at our institution between July 2014 and December 2018. The diagnostic validity of implant sonication was analyzed on the basis of the last proposed definition criteria of the International Consensus Meeting and compared with standard tissue cultures.	Periprosthetic tissue culture (PTC).	Of the 28 infected patients, 20 (71.4%) had an identified organism by tissue cultures, and *Cutibacterium acnes* was the most commonly isolated pathogen. The sensitivities of sonicate fluid (≥50 CFU/mL) and periprosthetic tissue culture for the diagnosis of periprosthetic shoulder infection were 36% and 61% (P = 0.016), and the specificities were 97.7% and 100% (P > .99), respectively. If no cutoff value was used in sonication culture, the sensitivity increased to 75% whereas the specificity dropped to 82%. Although there was no significant difference in sensitivity between tissue culture and the no-cutoff sonication fluid culture (61% vs. 75%, P = .125), the specificity of tissue culture was significantly higher (100% vs. 82%, P = .01).
(Hoekstra et al., 2020)	Retrospectively study.	Assess the clinical importance of a standardized sonication protocol in detecting PJI.	All patients with revision surgery of a hip or knee prosthesis between 2011 and 2016 were retrospectively reviewed and divided in two groups: clinically suspected of infection or not suspected of infection. For both tissue culture and implant sonication, calculations of sensitivity and specificity were performed. Clinical relevance of sonication was evaluated by calculating in which percentage of patients' sonication influenced clinical treatment.	Periprosthetic tissue culture (PTC).	Sensitivity of perioperatively taken tissue cultures was 94.3% and specificity was 99.3%. For sonication sensitivity was 80.5% and specificity was 97.8%. In the infection group eight patients (9%) with only one positive tissue culture and a positive sonication fluid culture with the same pathogen were found.
(Torrens et al., 2020)	Retrospectively study.	Determine whether sonication yields greater sensitivity when compared with the traditional tissue culture in detecting periimplant infections in shoulder surgery.	Includes 99 shoulder surgeries with implants explanted. The inclusion criteria required at least four tissue cultures, sonication of the material explanted, and a minimum follow-up of two years. Patients were classified according to the definition of periprosthetic shoulder infection of the ICM 2018 on Orthopedic Infections. The classifications are definitive infection, probable infection, possible infection, and unlikely infection.	Periprosthetic tissue culture (PTC).	Considering the cases with a definitive infection, the sensitivity of the tissue culture was 87.09% and the sensitivity of sonication stood at 80.64% (p = 0.406). Analyzing the cases with a definitive infection and those having a possible/ probable infection together and comparing them with those with unlikely infection, the sensitivity of sonication was 80.4% and the sensitivity of the tissue culture came to 91.4%. The specificity of the sonication was 98.1% and the specificity of the tissue culture was 99.6%.
(Randau et al. 2021)	Retrospectively study.	Assess the performance of a commercially available dithiothreitol (DTT) kit for routine use in diagnosing PJIs in comparison to conventional microbiological tissue specimens and sonication procedures in a maximal care hospital.	Applied the DTT system in 40 consecutive cases of revision arthroplasty (23 PJIs and 17 aseptic revisions), with an exchange or a removal of components. The hardware components were split between the DTT system and the conventional sonication procedure. At least three tissue biopsies and a joint fluid specimen were sent for microbiological and histopathological analysis.	Dithiothreitol (DTT)	Cultures of the DTT fluid showed a sensitivity of 65% and specificity of 100%, as referenced to conventional microbiological cultures. Sonication had better sensitivity (75%) but lower specificity (85%). The categorical agreement of DTT cultures compared to sonication fluid cultures was 78% (31/40).
(Rieber et al., 2021)	Retrospectively study.	Analyze the accuracy of our culture techniques for the diagnosis of PJI.	Tissue samples and components from 258 patients after revision arthroplasty of the hip, knee, and shoulder were investigated, and the results of TC were compared to those of SFC. Furthermore, an evaluation was performed of the influence of different culture media on the detection rate.	Periprosthetic tissue culture (PTC).	The overall sensitivity of TC was no different to that of SFC (91.3% vs 90.8%, P = 1). In 153 cases (82.3%), TC and SFC showed concordant positive results. Results were discordant in 33 cases (17.7%). When differentiated according to the type of infection, TC showed significantly better results than SFC in detecting polymicrobial infections (97.0% vs 67.0%, P = 0.004).
(Stephan et al., 2021)	Retrospectively study.	Assess the influence of preoperative antibiotic prophylaxis (PAP) and antibiotic therapy (AT) on sonicated fluid cultures in patients with implantassociated infection compared to conventional tissue culture.	Three groups were compared: (I) standard PAP, (II) AT for at least one day, and (III) no antibiotics before surgery. For the inclusion criteria, an established diagnostic protocol for implantassociated infection was used. Sonicate fluid cultures were validated by corresponding microbiological and histopathological samples.	Periprosthetic tissue culture (PTC), in three different groups: (I) standard PAP, (II) AT for at least one day, and (III) no antibiotics before surgery.	The detection rate by sonicate fluid cultures in patients receiving PAP (n = 27, 29 pathogens), AT before surgery (n = 33, 48 pathogens) and no antibiotics before surgery (n = 30, 37 pathogens) were 86.2%, 81.3%, and 86.5% (p = .778), respectively. Eleven of 114 infectious agents were detected exclusively by sonicate fluid cultures, while conventional tissue culture failed in these cases.
(Flurin et al., 2021)	Retrospectively study.	Assess sonication for PJI diagnosis after Total Elbow Arthroplasty (TEA).	Retrospectively analyzed 112 sonicate fluid cultures from patients who underwent revision of a TEA at a single institution between 2007 and 2019, comparing results to those of tissue cultures. Excluded patients who had fewer than 2 tissues submitted for culture. Used the Infectious Diseases Society of America guidelines to define PJI. In addition, compared the sensitivity of tissue culture to the combination of tissue and sonicate fluid culture.	Periprosthetic tissue culture (PTC).	The most common pathogens were coagulase-negative *Staphylococcus* sp (49%), followed by *Staphylococcus aureus* (12%). Sensitivity of tissue culture was 63%, and sensitivity of sonicate fluid culture was 76% (P = .109). Specificity of tissue culture was 94% and specificity of sonicate fluid culture was 100%. Sensitivity of sonicate fluid culture in combination with tissue culture was 84% (P = .002 compared to tissue culture alone).
(Sebastian et al., 2021)	Prospective study.	Evaluate the diagnostic utility of DTT treatment of periprosthetic tissue and explanted implants, as compared to the normal saline treatment of periprosthetic tissues and sonication of explanted implants for the diagnosis of PJI.	Seventy-three revision arthroplasty cases were prospectively included in this study. Three to five tissue specimens and the explanted implants were collected from each patient. Periprosthetic tissue samples were processed by both normal saline and DTT treatments. Explanted implants were subjected to both DTT treatment and sonication.	Dithiothreitol (DTT).	The sensitivity of DTT treated periprosthetic tissue culture (PTC) and saline treated PTC was similar (66.6% vs 58.8%, P = 0.25). The specificity of both was 100%. Sonication and DTT treatment of explanted implants showed comparable sensitivity (85.3% vs 82.4%) and specificity (100% vs 97.4%), P > 0.99. Compared to DTT treated PTC, culture of DTT treated explanted implants significantly improved the diagnosis of PJI (P = 0.03).
(Aliyev et al., 2022)	Prospective study.	Evaluate the sensitivity and specificity of the sonication cultures and to evaluate the effect of sonication on the antibiotic treatment of patients.	Sixty-four patients who were scheduled for revision hip or knee arthroplasties were included in the study. Aspiration fluid, tissue, and sonication cultures were performed from all patients and compared in terms of sensitivity, specificity, positive predictive value (PPV), negative predictive value (NPV), and overall accuracy. Other targets of the study were to investigate the rate of change in the antibiotic treatment.	Aspiration fluid and periprosthetic tissue cultura (PTC).	The sensitivity, specificity, PPV, NPV, and overall accuracy of the fluid culture obtained by the sonication method were 71.4%, 96.6%, 96.2%, 73.7%, and 82.8%, respectively. The sensitivity, specificity, PPV, NPV, and overall accuracy of the fluid culture obtained after tissue sampling were 68.6%, 100%, 100.0%, 72.5%, and 82.8%, respectively. There was no statistically significant difference between the sonication method and tissue culture in terms of sensitivity and specificity (p = 1.0). The sensitivity, specificity, PPV, NPV, and overall accuracy of the fluid culture obtained by the aspiration method were 28.6%, 93.1%, 83.3%, 51.9%, and 57.8%, respectively.
(Karbysheva et al. 2022)	Prospective study.	Compare the biofilm dislodgement efficacy of chemical method (DTT) compared to the sonication procedure in the diagnosis of PJI.	187 patients undergoing hip and knee prostheses explantation were included, of whom 94 were assigned for sonication and 93 for DTT group.	Dithiothreitol (DTT).	Sonication demonstrated superior sensitivity (73.8%) compared to DTT (43.2%) in diagnosing PJI, with comparable specificity levels (98% and 94.6%, respectively).

The 12 selected articles are detailed by author, type of study, objective of the study, methodology, comparator and tabulated in ascending order according to the year of publication.

After conducting an exploratory reading of the material obtained, the following points were discussed: 1- the sonication protocol used and the results obtained regarding the sensitivity and specificity compared with those of periprosthetic tissue culture; 2- the main microorganisms isolated; and 3- the ability of sonication protocols to displace biofilm structure compared to other displacement techniques.

## 3 The sensitivity and specificity of sonication protocols are greater than those of periprosthetic tissue culture

Differences in the parameters of the sonication protocols adopted in the selected studies were observed. These differences include the use and duration of vortexing, the use of centrifugation as a method for determining sample concentration after vortex agitation, and variations in the sonication bath concerning frequency, power density, and time. Additionally, cutoff values for microbial count to define infection differed among the studies (**Table 2**).

**Table 2:** Characterization of the studies employing sonication.

Author/Year	Number of samples/patients	Type of infection	Infection Criteria	Vortex use	Sample centrifugation	Sensitivity and Specificity of SFC and TC	CFU cut off points (culture positivity)
(Ueda et al., 2019)	493 cultures.	Orthopedic ImplantAssociated Infection (OIAI)	Musculoskeletal Infection Society (MSIS).	Yes Before and after sonication	Yes	Sonication: 70.6% (44.0-88.6 CI 95%)/100% (91.1-100 CI 95%). Tissue culture: 58.8% (33.5-80.6 CI 95%)/98.0% (88.099.9 CI 95%).	1 CFU/plate.
(Flurin et al., 2021)	112 patients.	49 with PJI and 63 with aseptic failure	Infectious Diseases Society of America (IDSA).	Yes Before sonication	No	Sonication: 76%/100% Tissue culture: 63%/94%.	≥ 20 CFU/mL, except for virulent microorganisms such as *S. aureus*, for which any growth was considered positive.
(Bellova et al., 2019)	257 of potentially infected prostheses	Periprosthetic joint infection (PJI)	European Bone and Joint Infection Society (EBJIS) and International Consensus Meeting (ICM) criteria.	No	No	EBJIS criteria: Sonication: 69.0% (*p* = 0.04)/100% (*p* = 0.65). Tissue culture: 63% (*p* = 0.04)/94% (*p* = 0.65). ICM criteria: Sonication: 87,5% (*p* = 0.63)/85.1% (p=0.05). Tissue culture: 84,4% (*p* = 0.63)/92.5% (*p* = 0.05).	>50 CFU/mL, except for virulent microorganisms such as *S. aureus* and anaerobes for which any growth was considered positive.
(Rieber et al., 2021)	258 patients	Periprosthetic joint infection (PJI).	Musculoskeletal Infection Society (MSIS) updated by Parvizi et al., 2018 (Parvizi et al., 2018)	Yes Before and after sonication	No	Sonication: 90.8% (*p* = 1) Tissue culture: 91.3% (*p* = 1).	Isolation of the same organism by culture from two or more separate tissue or fluid samples from the prosthesis.
(Akgün et al., 2020)	80 patients	Periprosthetic joint infection (PJI) of shoulder	International Consensus Meeting (ICM).	Yes Before and after sonication	No	Sonication: 36% (*p* = 0.016)/97.7% (*p* > 0.99). Tissue culture: 61% (*p* = 0.016)/ 100% (*p* > 0.99).	Sonication: ≥ 50 CFU/mL of a low virulent organism or any growth of a high-virulent organism was present. Tissue culture: ≥ 2 CFU/mL.
(Torrens et al., 2020)	99 shoulder surgeries with implants explanted	Periprosthetic Joint Infection (PJI) of shoulder	International Consensus Meeting (ICM).	Yes Before and after sonication	No	Sonication: 80.04% (*p* = 0.406)/98.1% (*p* = 0.027) Tissue culture: 87.09% (p = 0.406)/99.6% (*p* =0.027).	50 CFU/mL.
(Hoekstra et al., 2020)	226 patients	Periprosthetic Joint Infection (PJI) of Hip or Knee.	International Consensus Meeting (ICM).	Yes Before and after sonication	No	Sonication: 80,5% (71-88 CI 95%)/97.7% (94-99). Tissue culture: 94,3% (87-98 95% CI)/ 99,3% (96-99 95% CI).	Any growth observed in the sonication fluid that were not contaminants, based on the discretion of the attending clinical microbiologist, was considered positive.

The methodology of the studies selected in topic 1 is outlined in **Table 2**, with a focus on the parameters employed in their sonication protocols.

The studies analyzed also calculated the sensitivity and specificity percentages of their sonication protocols and standard cultures. Sensitivity is defined as the ability of the diagnostic test to detect individuals who are truly positive and is calculated according to the number of true positives divided by the number of true positives added to the number of false negatives (TP/(TP+FN)), using the gold standard test as a reference. Specificity is defined as the ability of the diagnostic test to detect true negatives and is calculated according to the number of true negatives divided by the number of true negatives plus the number of false positives (TN/(TN+FP)) using the gold standard test as a reference (Ueda et al., 2019).

The first analyzed sonication protocol included vortex mixing of the container with the implant immersed in sterile saline solution for 30 s, an ultrasound bath at a frequency of 40 ± 2 kHz, and 0.22 ± 0.04 W/cm^2^ for 1 min, followed by vortexing for another 30 s. Then, 50 mL of sonicated fluid was centrifuged at 2600 rpm for 15 minutes and cultured. The cutoff for a positive result was ≥1 CFU/plate, calculated as CFU/mL based on CFU/plate. For statistical tests, 2x2 contingency tables were constructed consisting of true-positive (TP), false-positive (FP), false-negative (FN) and true-negative (TN) results, taking positive results for the disease as a reference according to MSIS criteria. Ninety-five percent confidence intervals were calculated as exact binomial confidence intervals. The sensitivity and specificity of the different diagnostic culture methods were compared by McNemar’s test of paired proportions. All testing was conducted using SPSS v22.0 software (SPSS, Inc., Chicago, IL), with a *p* value < 0.05 (in 2-sided testing) considered to indicate statistical significance (Ueda et al., 2019).

The reported sensitivity for SFC was 71%, 95% CI (44.0-88.6), while PTC achieved a sensitivity of 59%, 95% CI (33.5-80.6) at a cutoff point of 1 colony-forming unit/plate and 1 positive culture. Furthermore, the detection rate of orthopedic implant-associated infection (OIAI) attributed to sonicated fluid culture was significantly greater than that attributed to tissue culture (61% vs. 36%; *p* = 0.02). Using the cutoff point of 2 positive culture, the combination of the two methods (PTC and SFC) showed better sensitivity than the conventional method (94%, 95% CI (69.2-99.7) vs. 82%, 95% CI (55.8-95.3); *p* = 0.25) (Ueda et al., 2019).

A second study used the sonication protocol proposed by Trampuz et al., 2007 (Trampuz et al., 2007). The container with the prosthetic components was filled with Ringer’s solution (an isotonic solution containing sodium, chloride, potassium, calcium and sodium lactate used to prevent osmotic shock in bacteria in procedures intended for the preparation of suspensions), vortexed (30 s), sonicated at a frequency of 40 ± 2 kHz and 0.22 ± 0.04 W/cm^2^ (Aquasonic Model 750T - VWR Scientific Products) for 5 min, vortexed for an additional 30 s, and then cultured. Sensitivities and specificities were also calculated using a 2×2 contingency table for both methods, as well as their 95% confidence intervals. To compare the sensitivities and specificities of the different tests, the McNemar test was used to compare paired proportions (*p* value < 0.05) (Flurin et al., 2021).

For sonicate fluids, was considered a culture positive if there was growth of greater than 20 CFU/10 mL of sonicate fluid, with the exception of virulent microorganisms such as *S. aureus*, for which any growth was considered positive. The sensitivity and specificity of SFC were 76%, 95% CI (62-85), and 100%, 95% IC (94-100), respectively, while for PTC, these values decreased by 63%, 95% CI (49-75), and 94%, 95% IC (85-98), respectively. The sensitivity of both tests combined (84%, 95% CI 71-91) was significantly greater than the sensitivity of tissue culture alone (63%, *p* = 0.002) (Flurin et al., 2021).

In another study, the explanted prostheses were immersed in Ampuwa^®^ solution (a highly pure hypotonic water that does not contain dissolved substances) followed by an ultrasonic bath for 1 min at 80% power (P=160 W) (BactoSonic; Bandelin, Berlim, Alemanha), and no vortex was used before culture. Sensitivity and specificity were determined (2x2 contingency tables for SFC and PTC and their proportions calculated using the McNemar test, using SPSS software - IBM Corporation; Armonk, NY, United States) for the diagnosis of PJI defined according to the EBJIS criteria, which considers a count of > 50 CFU/mL of any organism as positive for PJI, and in accordance with the criteria of the ICM 2018, which considers two positive cultures of the same organism as the major criterion for the diagnosis of PJI and a single positive culture as the minor criterion (Bellova et al., 2019).

Based on the EBJIS infection criteria, there was a statistically significant difference in sensitivity between sonication fluid culture and tissue culture (*p* = 0.04). SFC exhibited a sensitivity of 69.0% (100/145 patients were accurately identified by the SFC as positive) and a specificity of 90.2% (101/112 patients were accurately identified by the SFC as negative), while PTC demonstrated a sensitivity of 62.8% (91/145) and a specificity of 92.9% (104/112). However, when the ICM 2018 criteria were adopted, the sensitivities of SFC and PTC were 87.5% (84/96) and 84.4% (81/96) (*p* = 0.63), respectively, while the specificities were 85.1% (137/161) and 92.5% (149/161) (*p* = 0.05), respectively (Bellova et al., 2019).

On the other hand, even when using a standardized protocol (Trampuz et al., 2007) that has already shown positive results (Flurin et al., 2021), the general sensitivity obtained was not significantly different between PTC and SFC (91.3%, 170/186 vs 90.8%, 169/186; *p* = 1), considering isolation of the same organism by culture from two or more separate tissue or fluid samples from the prosthesis as positive. However, examining the results based on infection type, PTC demonstrated better performance in detecting polymicrobial infections than did SFC (97.0%, 32/33 vs 67.0%, 22/33; *p* = 0.004), as determined by the two-proportion Z test using RStudio software (version 1.2.5042) (*p* < 0.05) (Rieber et al., 2021).

Better results for PTC were also observed using a proposed sonication protocol (Renz et al., 2018) that included the addition of saline solution to the container with the prosthesis to cover most of the implant, then an initial vortex shaking (30 s) of the container, followed by a sonication bath for 1 min at 40 kHz (BactoSonic; Bandelin Electronic, Berlin, Germany), and another 30 seconds of vortex mixing. A cutoff point of ≥ 50 CFU/mL was used to determine positivity by sonication of the fluid and isolation of the same organism from 2 or more tissue samples to determine positivity by standard culture (ICM 2018) (Akgün et al., 2020).

Using a 2x2 contingency table, McNemar comparison test, chi-square test and Fisher’s exact test to determine significant differences between categorical variables (*p* < 0.05) (SPSS version 20 - IBM, Armonk, NY, USA) resulted in a sensitivity of 36% for SFC (≥ 50 CFU/mL), while it was 61% for PTC (≥ 2 tissue samples with the same organism) (*p* = 0.016). The specificity was 97.7% for SFC and 100% for PTC (*p* > 0.99). However, when the cutoff value was eliminated in sonication culture, the sensitivity increased to 75%. Nevertheless, this increase in sensitivity came at the expense of decreased specificity, which decreased to 82%. These changes did not result in a statistically significant difference in the diagnostic benefits of SFC compared to PTC (Akgün et al., 2020).

Similar findings were reported when assessing the sensitivity of SFC compared to that of PTC in detecting peri-implant infections in shoulder surgery using a similar reported protocol (Akgün et al., 2020). After the removed components were transported in a polyethylene container with approximately 200–400 ml of sterile saline, the container was first vortexed for 30 seconds and then sonicated for one minute at a frequency of 40 ± 5 kHz in a Bransonic^®^ SM25E-MT ultrasound bath (Branson Ultrasonics Corporation, Geneva, Switzerland) after vortexing again for 30 seconds. Sonication was considered positive if at least 50 CFU/mL was detected (ICM 2018). PTC achieved 87.09% sensitivity, and SFC reached 80.64% (*p* = 0.406). The specificity of PTC was 99.6%, and that of SFC was 98.1% (*p* = 0.175) (*p* < 0.05) (sensitivity, specificity, ROC area and Delong comparison test calculated in STATA 15.1). No statistically significant difference was found between the results obtained by the two methods (Torrens et al., 2020).

The sonication procedure, which involved immersing the prosthetic container (90%) in Ringer’s solution, followed by 30 seconds of shaking, a 1-minute sonication bath at 100% power (200 W, 0.22 W/cm^2^) (Bandelin Bactosonic), and another 30 seconds of shaking before fluid culture, also yielded favorable results for PTC compared to SFC. Using this protocol and ICM 2018 criteria, the PTC sensitivity was 94.3%, 95% CI (87-98), and the specificity was 99.3%, 95% CI (96-99). The sensitivity for SFC was 81%, 95% CI (71-88), and the specificity was 97.8%, 95% CI (94-99), which were considerably lower than the results observed for PTC (2x2 contingency table using SPSS version 22.0). Although the sensitivity and specificity of SFC were lower than those of PTC, it is worth noting that 8 patients (9% of the total) suspected of having a periprosthetic joint infection could be definitively diagnosed based on a positive result from SFC (Hoekstra et al., 2020).

Sonication has shown variable diagnostic accuracy in these studies. It was possible to observe that the best sensitivity and specificity indices, compared to those of PTC, were achieved by sonication protocols that used sterile saline solution for immersion of the prosthesis, low frequencies of ultrasound waves (40 kHz) for a period of 1 or 5 minutes, the use of a vortex (before and after sonication) and centrifugation and even lower cutoff points (≥1 CFU/mL) than the 50 CFU/mL recommended by some consensuses.

In general, the results obtained with SFC were better for diagnosing PJI. Even in some studies in which its sensitivity and specificity were lower than those of PTC, it was possible to observe a small significant difference or even no significant difference. This superiority is magnified when we analyze the cost-benefit of the technique, which presents potential improvement in culture results, in a simple technique with lower recurrence rates (associated with the diagnostic inaccuracy of traditional tissue culture) and costs. Therefore, it is easily applicable in clinical practice from surgical and microbiological points of view (Flurin et al., 2021). However, considering the positive contribution that PTC can add to the diagnosis (Rieber et al., 2021), it is still possible to perform a combination of SFC and PTC (Ueda et al., 2019).

## 4 Microorganism detection capacities of SFC and PTC and the main microorganisms isolated

Among the predominant microorganisms identified in the OIAI, coagulase-negative *Staphylococcus* species (CoNS) were the primary causative agents of infections in 24 isolates detected by both the SFC and PTC methods. However, 18% of the positive diagnoses were exclusively identified using sonication. In these cases, less virulent species, such as *Streptococcus* of the *viridans* group, *Peptostreptococcus*, and *Corynebacterium* spp., were also isolated. *Peptostreptococcus* and *Corynebacterium* spp. were isolated by SFC only. Among patients who had prior antibiotic therapy, 67% of those who received SFC had infections (Ueda et al., 2019).

In a recent study, the most common pathogen isolated from periprosthetic elbow infection using both methods was CoNS (49%), followed by *Staphylococcus aureus* (12%), gram-negative *Enterobacter cloacae* (3%) and *Klebsiella pneumoniae* (2%). The authors observed that among the positive cultures, 78% exhibited monomicrobial cultures, while 22% had polymicrobial cultures. The SFC method played a crucial role in identifying the majority of polymicrobial infections, leading to treatment modifications in 4 out of 5 patients. However, it is worth noting that SFC failed to detect *Corynebacterium amycolatum*, a species that was only identified through tissue culture, but it did not have any impact on the choice of antibiotic regimen. In 10 patients, only sonicate cultures were positive: 7 for *S. epidermidis*, 1 for coagulase-negative *Staphylococcus* sp., 1 for *S. aureus* and 1 for *Parvimonas micra* (Flurin et al., 2021).

In cases of PJI, the primary pathogens were also CoNS. The SFC method successfully isolated 38 of these microorganisms, whereas the PTC method yielded only 30 isolates. The second most frequently isolated microorganism was *S. aureus*, which was further classified as *methicillin-susceptible Staphylococcus aureus* (MSSA) *or methicillin-resistant Staphylococcus aureus* (MRSA). There were more SFC isolates than PTC isolates for both MSSA (14 vs. 12) and MRSA (5 vs. 4). They also observed that in early PJI detected using sonication, 48.9% of the cases were attributed to high-virulence pathogens, while 51.1% were associated with low-virulence pathogens. A similar pattern was observed for delayed and late infections combined, with 35.7% classified as having high virulence and 64.3% as having low virulence. Significantly, 7.8% of delayed or late infections detected using SFC were positive for anaerobes, with *Cutibacterium acnes* identified as the predominant species. In comparison, only 2.6% of infections were detected through PTC (Bellova et al., 2019).

In addition, for the microbiological diagnosis of PJI, 220 microorganisms were isolated from the PTC and SFC methods, and concordant positive results were obtained for 153 out of 186 patients (82.3%). The CoNS (n= 60) were also the main group of bacteria isolated, with *Staphylococcus epidermidis* (n = 43) as the main species. The second most prevalent pathogen was *S. aureus* (n= 55), followed by anaerobic bacteria (n=43), and members of the Enterobacterales family (n= 27), *Streptococcus* spp. (n= 17) and *Enterococcus* spp. (n= 14) were also isolated in this study (Rieber et al., 2021).

However, in periprosthetic elbow infections, the most frequently isolated pathogen by tissue culture was *C. acnes*, accounting for 46% of the cases. The second most isolated pathogen was CoNS, accounting for 17.9% of the cases, followed by *S. aureus*, accounting for 7%. Other bacteria isolated included *Finegoldia magna* (3.6%), *Streptococcus agalactiae* (13.6%), *Enterococcus faecalis* (3.6%), and *Peptoniphilus asaccharolyticus* (3.6%). In 75% of patients, at least one organism was successfully isolated by sonication. However, it is noteworthy that there were discordant results between SFC and PTC in 32% of the patients (Akgün et al., 2020).

This microbial identification was also observed in patients with confirmed infection in hip or knee prostheses. Among these patients, only eight individuals (9%) had positive cultures for the same pathogen using both the SFC and PTC methods. Certain pathogens could only be identified using the SFC method. These pathogens are typically low in virulence and are known to produce biofilms, making them particularly difficult to detect. Examples of such pathogens include *Streptococcus mitis*, *S. epidermidis*, *Aggregatibacter* species, *C. acnes*, and *Corynebacterium striatum* (Hoekstra et al., 2020).

Considering that peri-implant infections in the shoulder were definitively diagnosed, *S. epidermidis* was present in 42% of the patients, followed by *C. acnes* in 22.5%. Among these cases, 22.6% were classified as polymicrobial infections, with *C. acnes* being involved in most of these cases (71%) (Torrens et al., 2020).

When evaluating the influence of preoperative antibiotic prophylaxis (PAP) and antibiotic therapy (AT) on SFC in patients with implant-associated infections, 114 important infectious agents were detected, 11 of which were detected exclusively after the use of SFC. The main microorganisms isolated included CoNS, *S. aureus*, *Streptococcus* spp., and *Enterococcus* spp. Microorganisms were identified despite prior antibiotic therapy; therefore, they do not recommend omitting antibiotic prophylaxis in patients with implant‐associated infections (Stephan et al., 2021).

Moreover, although the SFC technique did not enhance the sensitivity of microbiological diagnosis for PJIs in this study, it did demonstrate the ability to identify distinct microorganisms compared to other methods. This finding contributed to changes in the strategy of antibiotic therapy for infected patients, as it relies on antimicrobial sensitivities derived from microbiological culture results (Aliyev et al., 2022).

Sonicated fluid culture plays an important role in the detection of particular microorganisms, such as *Peptostreptococcus, Streptococcus mitis, S. epidermidis, Aggregatibacter* species, *Corynebacterium* spp. and *Cutibacterium acnes*. C. acnes is responsible for chronic and low-grade infections that represent an additional challenge to the diagnosis of PJI, with an emphasis on *C. acnes*, which decreases the sensitivity of traditional diagnostic tests for infections associated with orthopedic implants (Renz et al., 2018; Hoekstra et al., 2020). In addition to the aforementioned bacteria, fungal PJI, although rare (1% to 2%), can be difficult to control and identify because the isolation of organisms by traditional culture can take a long time, resulting in false negatives (Chisari et al., 2022). It is believed that fungi and mycobacteria are responsible for more than 85% of cases of negative cultures in PJI (7%-15%). In this context, sonication is a low-cost method capable of increasing the chances of identifying the causative agent (Palan et al., 2019).

The ability of CFS to identify diverse and especially low-virulence microorganisms, even in the face of preoperative antibiotic prophylaxis, can affect the antibiotic therapy strategy adopted (Aliyev et al., 2022). This characteristic has the potential to increase the effectiveness of treatment, reduce costs associated with prolonged use of antibiotics and longer hospital stays, often requiring multiple surgical procedures, thus reducing unnecessary exposure to antibiotics; consequently, bacterial resistance has increased dramatically in the last ten years, probably due to the excessive and often inappropriate use of antibiotics (Drago and De Vecchi, 2017).

This ability may further permit the use of an antibiotic-loaded bone cement spacer sonication fluid culture technique to confirm the eradication of infection or two-stage revision reinfection prior to reimplantation of new prostheses. It can be used accurately as a complement to evaluate the therapeutic effect of IAP (Zhang et al., 2021a).

## 5 The ability of sonication protocols to dislodge biofilm structure compared to that of other displacement methods

Dislodging of bacterial cells from the biofilm structure can be achieved by mechanical means or chemical or physical methods. Mechanical methods such as scraping the prosthesis or vortices have rarely been evaluated; when studied, they have shown low performance (Bjerkan et al., 2009; Drago and De Vecchi, 2017), and in the current literature, they are scarce (Drago and De Vecchi, 2017). The use of chemical substances in explanted implants and periprosthetic tissues is suggested as a possible biofilm dislodgement method with possible applicability in the diagnosis of infections associated with implants. Among the proposed agents are the metal chelator ethylenediaminetetraacetic acid (EDTA) and the strong reducing agent dithiothreitol (DTT) (Karbysheva et al., 2020).

It was suggested that the activity of EDTA against biofilm cells occurs through the chelation of magnesium, calcium, and iron, enhancing the detachment of cells from the biofilm matrix (Banin et al., 2006). Additional observations included that the mean colony count (logCFU/mL) after DTT treatment was comparable to that achieved after sonication or physical methods and greater than the count obtained using the scraping technique (Drago et al., 2012).

Compared with DTT treatments for the diagnosis of PJI, the explanted implants were immersed in 0.1% w/v TDT (Promega, Madison, WI, USA) in sterile saline and kept in an incubator with shaking for 15 minutes at room temperature, followed by centrifugation at 3,000 rpm for 10 minutes before standard cultivation. With a sonication protocol for explanted implants, 90% of the prosthesis was immersed in sterile saline solution, followed by vigorous manual shaking for 30 seconds before and after the sonicated bath, which was programmed for 7 min at 40 kHz in BactoSonic (Bandelin GmbH, Berlin, Germany). The procedure was finished with centrifugation at 4,000 rpm for 20 minutes. Using the MSIS definition of IAP, both methods, SFC and DTT, demonstrated similar sensitivity rates of 85.3% (29/34) and 82.4% (28/34) (*p* > 0.05) (analysis performed with STATA software version 14.2 - Stata Corp LLC; Texas, USA), respectively. Although not statistically significant, the specificity was greater when using the SFC technique (100%, 39/39 vs. 97.4%, 38/39 *p* > 0.99) (Sebastian et al., 2021).

In another study, DTT treatment involved the use of a MicroDTTect closed system for biofilm processing via this chemical method. Explant samples were collected from the sterile system itself, and then, in the laboratory, the chamber valve containing TDT (150 mL, 0.1% p/v) was broken, allowing DTT to flow into the explant. The device was subsequently mechanically stirred for 15 minutes at room temperature, after which standard cultivation continued. For the sonication protocol, the explanted implants were collected in sterile plastic bags that were subsequently filled with sterile saline solution, vortexed for 30 seconds and sonicated in a Bactosonic 14.2 device (Bactosonic, Bandelin, Berlin, Germany) for 5 minutes with a frequency of 40 ± 2 kHz and a power density of 0.22 ± 0.04 W/cm2 followed by 30 seconds of vortexing (Randau et al., 2021).

Using the MSIS criteria to define PJI, the authors found a sensitivity of 65% (13/20) and specificity of 100% (20/20) for DTT fluid culture compared to conventional microbiological cultures. Sonication had better sensitivity (75%, 15/20) but lower specificity (85%, 17/20) than conventional microbiological culture (*p* > 0.05) (statistical significance between groups was assessed by the Mann-Whitney test). Fisher’s exact test was used for contingency, sensitivity and specificity analysis. The analysis was performed using GraphPad Prism 8.0.2 (GraphPad Software, La Jolla, CA, USA). The categorical concordance of DTT cultures with that of SFC cultures was 78% (31/40) (Randau et al., 2021).

Based on these results, sonication has been shown to be the main assay for biofilm detection in the microbiological diagnosis of implant-associated infection (Karbysheva et al., 2020). Even in one study that showed a loss of specificity, sonication provided a more reliable diagnosis of PJI, as it identified more pathogens than DTT treatment (Karbysheva et al., 2022). However, given the positive impacts that chemical methods can have on the diagnosis of these infections, especially on culture specificity, DTT treatment could be used as a biofilm displacement technique in situations where sonication is not viable or possible (Sebastian et al., 2021). It is also possible to evaluate the potential additive effect of chemical shift on sonication (Karbysheva et al., 2020).

## 6 Proposal for a standardized sonication protocol

**Figure 1:**
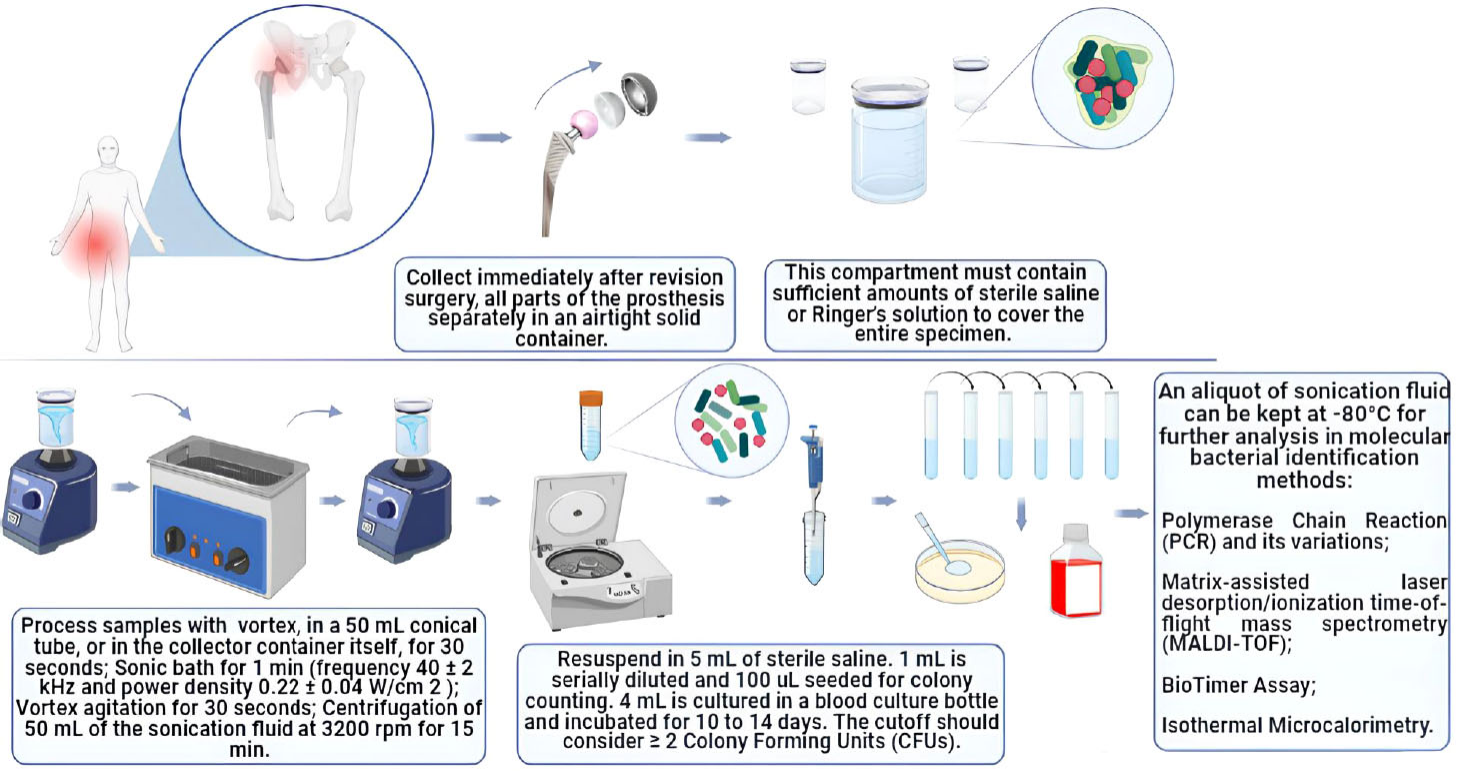
Diagram of the sonication protocol according to the parameters reviewed in this study.

To propose the best parameters for establishing a sonication protocol, studies by Cieslinski et al., 2021, Trampuz et al., 2006, Oliva et al., 2021, Rosa et al., 2019, Ueda et al., 2019, Dudek et al., 2020, Li et al., 2018, Ribeiro et al., 2022, Beguiristain et al., 2023, Borens et al., 2013 and Morgenstern et al., 2020 (Trampuz et al., 2006; Borens et al., 2013; Rosa et al., 2019; Ueda et al., 2019; Dudek et al., 2020; Morgenstern et al., 2020; Cieslinski et al., 2021; Oliva et al., 2021; Ribeiro et al., 2022; Beguiristain et al., 2023), were also reviewed in addition to the abovementioned studies (**Figure 1**).

### 6.1 Material collection

After revision surgery, it is recommended that all prosthetic components, periprosthetic cement or osteosynthesis devices, including polyethylene (PE) materials and metal and polymethylmethacrylate (PMMA) components, be carefully removed to avoid direct contact with the patient’s skin. These items should be collected separately. To ensure the integrity of the collected samples and preserve the viability of microorganisms, it is important to use airtight containers. It has already been demonstrated that storing samples in plastic bags can significantly reduce colony-forming unit (CFU) counts and is associated with the risk of contamination (Rosa et al., 2019; Cieslinski et al., 2021).

The use of plastic bags for storage promotes the desiccation of microorganisms, which can lead to changes in their biophysical properties, such as surface tension. This desiccation can disrupt physiological processes, including the growth of microorganisms (Cieslinski et al., 2021).

Thus, physical containers with thicker, completely sealed (hermetic) surfaces can serve as a protective measure by preventing water loss and helping to maintain microbial viability while reducing the risk of contamination. It is important for the entire sample to be covered, so these compartments should contain an adequate amount of sterile saline or Ringer’s solution. By ensuring a sealed and moist environment, physical containers can help preserve the viability of microorganisms and maintain their physiological state during storage. This is crucial for accurate microbiological analysis and for reducing the potential for false-negative results or alterations in microbial characteristics.

### 6.2 Sample storage

The samples should be processed soon after removal, ideally within 2 to 4 hours. However, if immediate processing is not feasible, the samples can be stored at -4°C without liquid. Refrigerated samples can be stored for 7 days, and although there may be a minor decrease in the bacterial load over time, this decrease is unlikely to have a significant impact on the culture’s positivity. This is particularly true when molecular methods are employed for bacterial identification of sonicated fluid (Cieslinski et al., 2021).

### 6.3 Vortex-sonication-vortex method

In this proposed protocol, the processing of the samples involves the following steps:

1. Sample vortex: The sample is vortexed for 30 seconds in the collection container, ensuring that the prosthesis is completely submerged in a sterile saline solution (the amount of solution depends on the sample size).
2. Sonication bath: The sample was immersed in a sonication bath for 1 min at a frequency of approximately 40 ± 2 kHz and a power density of 0.22 ± 0.04 W/cm^2^.
3. Vortex Agitation: After sonication, the sample was vortexed again for 30 seconds.
4. Centrifugation: Approximately 15 mL of sonication fluid (without the prosthesis) was centrifuged at 3200 rpm for 15 minutes to concentrate the sample (bacterial cells, probably present in the sonication fluid) for later culturing.

As a standard procedure to minimize the risk of contamination in subsequent protocol steps, it is important to change the water in the ultrasonic bath after each round of sonication.

The use of vortex stirring and centrifugation has been shown to contribute to enhancing specificity (Rosa et al., 2019; Oliva et al., 2021). With respect to the duration of sonication, a 1-minute duration produced good results. This short period of sonication helps to avoid potential bactericidal effects of the procedure (Ueda et al., 2019).

The ideal ultrasound frequencies for sonication to identify etiological agents of implant-associated infections, particularly PJI caused by pathogens such as *S. aureus*, *P. aeruginosa*, and *E. coli*, are 35 kHz and 40 kHz (Dudek et al., 2020). These frequencies were effective in displacing bacteria from biofilms, and they had a significant impact on the survival of bacteria, particularly those in a planktonic state.

The majority of studies describing the use of the sonication technique for explant prostheses use a sonication bath, often due to sample size constraints. However, it is worth noting that recently, an article demonstrated greater sensitivity with direct intraoperative sonication culture of implants and soft tissues than with conventional synovial fluid culture utilizing a portable probe sonicator (Shanghai Weimi Ultrasonic Co., Ltd.) (Ji et al., 2023).

### 6.4 Microbiological analysis

1- The sediment obtained from the centrifuged solution must be resuspended in 5 mL of sterile saline solution. 2- To determine the microbial cell count and viability, 1 mL of the resuspension was serially diluted 10 times at a ratio of 1:10, resulting in a total volume of 1 mL. Between 3-100 μL of the last three dilutions were plated on Mueller Hinton agar plates and incubated for 18-24 hours at 37°C to facilitate bacterial growth. 4- The remaining 4 mL of the resuspension was inoculated into blood culture bottles. The bottles are subsequently incubated for 10 to 14 days to facilitate the detection of slow-growing or fastidious microorganisms that might be present in the sample. In cases where there is clinical evidence of infection but standard microbiological cultures yield negative results, it is advisable to conduct additional fungal and mycobacterial cultures.

The advantages of inoculation into blood culture bottles have recently been highlighted. Among them are the increased sensitivity and specificity of culture, even in patients who received prior antibiotic therapy, due to the presence of antimicrobial removal systems and lytic agents in blood bottles that further promote the release of intracellular microorganisms. In this context, the use of blood culture bottles to inoculate joint fluids directly at the patient’s bedside can be valuable. Additionally, the application of an innovative version of the sonication culture method involves direct sonication of the retrieved implant and soft tissue, without a sonication tube, intraoperatively. This method utilizes a BACT/ALERT 3D blood culture system, contributing to the increased effectiveness of microbiological diagnosis for PJI (Drago et al., 2019; Ji et al., 2023).

The incubation period should be extended to up to two weeks to enhance the likelihood of identifying causative agents comprehensively. For instance, species such as *Staphylococcus* spp. are more likely to emerge during the initial week of incubation, while *Cutibacterium* spp. are typically detected during the second week (Oliva et al., 2021). Once the etiological agent is accurately identified, it becomes possible to prescribe the most suitable treatment. In regard to antibiotics, selecting the appropriate type and dosage is crucial. Notably, the cure rate for patients with culture-negative PJI is generally low (Li et al., 2018).

### 6.5 Bacterial phenotypic identification and quantification from sonication fluid

A positive culture result for sonication fluid was determined by a bacterial concentration ≥ 2 CFU/mL in ≥ 2 cultures. In this context, a monomicrobial PJI is considered if only one bacterial species grows above the cutoff in sonicated fluid cultures. Conversely, polymicrobial PJI is diagnosed if more than one species is isolated following the same criteria. Following a positive blood culture, phenotypic identification should be conducted using culture media supporting the growth of both aerobic and anaerobic bacteria. It is important to note that for diagnosis, the identified species are more relevant than the CFU count.

Some studies used cutoff points ≥ 1 and ≥ 5 CFU, as the sensitivity of sonicated liquid cultures can be significantly reduced, especially in patients who have received previous antibiotic therapy or still have chronic, low-grade infection. It is worth mentioning that the cutoff point of 50 CFU/mL, which is defended by most medical societies and widely used in clinical practice, may not be ideal in these cases, despite its ability to distinguish effective infections (Ueda et al., 2019; Oliva et al., 2021).

The growth of any virulent microorganism responsible for high-grade acute infections, such as *Staphylococcus aureus* and gram-negative bacilli, will also be considered. However, the growth of low-virulence microorganisms responsible for chronic and low-grade infections, such as coagulase-negative *Staphylococcus, Enterococcus* spp., *Corynebacterium* spp. and *Cutibacterium acnes*, in a single sample must be evaluated in conjunction with the patient’s clinical context (Drago et al., 2019; Romanò et al., 2019; Ueda et al., 2019; Oliva et al., 2021). This is where sonication presents one of its greatest advantages, which is its ability to more efficiently identify bacteria responsible for chronic, low-grade and difficult-to-detect infections, as previously mentioned, helping to improve the poor performance of conventional microbiological methods for identifying these pathogens (Renz et al., 2018; Hoekstra et al., 2020).

According to our suggested protocol, any growth detected in the sonicated fluid culture from patients who received antibiotics within two weeks prior to sample collection should be regarded as a positive result (Ueda et al., 2019; Oliva et al., 2021).

### 6.6 Bacterial identification by molecular methods

The sonication protocol, involving the use of a vortex-sonication vortex followed by CFU counts, may have several limitations. These include the inability to dislodge all adherent microorganisms in the biofilm and the potential for sonication to affect microbial viability, leading to inaccurate CFU counts. Molecular methods can be employed to address these limitations and contribute to the identification of difficult-to-cultivate bacteria, anaerobes, and noncultivable bacteria (Rosa et al., 2019).

These molecular methods include polymerase chain reaction (PCR), such as bacterial identification based on the amplification of 16S ribosomal RNA, and various methods, such as multiplex PCR (mPCR), which can amplify the genetic material of different targets in a single process, thus allowing bacterial identification, as performed in commercial panels of multiplex PCR for IAP (Schoenmakers et al., 2023). It exhibits good sensitivity and requires less sample material and time than culture-based methods. Broad-range PCR can identify the predominant bacterial strain at infection sites of various cultural origins, even in patients undergoing antibiotic therapy. The main limitations of PCR-based diagnosis include the inability to discriminate between live and dead bacteria and DNA contamination. However, when used in conjunction with sonication, it has great diagnostic value for PJI, especially for routine clinical practice when used in panels, as already mentioned (Liu et al., 2018; Schoenmakers et al., 2023; Tsikopoulos and Meroni, 2023).

Another method is identification by matrix-assisted laser desorption/ionization time-of-flight mass spectrometry (MALDI-TOF), which allows direct identification of aerobic and anaerobic bacteria from positive blood cultures. MALDI-TOF has been successfully employed for detecting microorganisms in biological samples, whether from colonies or fluids. Several studies support the feasibility of using this technique for bacterial identification in sonicated fluid as well as direct identification in blood culture bottles. This approach facilitates early and reliable identification, serving as an alternative to culture methods (Cieslinski et al., 2021; Ribeiro et al., 2022; Beguiristain et al., 2023).

As additional techniques, we can also perform fluorescence *in situ* hybridization (FISH), in which fluorescent probes bind to complementary nucleic acid sequences to identify the presence or absence of these target sequences. The ability to identify bacteria in negative cultures reduces false positives through better identification of environmental contamination and the ability to exclude dead bacteria with a viability stain. Another technique that can help with pathogen identification is DNA microarrays, where microarrays allow the simultaneous measurement of large numbers of genes involving thousands of microscopic DNA sequences (probes) complementary to specific gene fragments of the microorganisms studied. However, both of these methods have the disadvantages of high cost, the need for specialized equipment, the potential for contamination and a lack of probes relevant for diagnosing PJI (Shoji and Chen, 2020).

We can also mention the use of identification methods based on specific bacteriophages for the pathogens studied, where DNA detection by qPCR and adenosine triphosphate (ATP) detection are performed after bacteriophage lysis. This technique aims to contribute to the development of a faster, more sensitive, specific and, at the same time, economical and practical system to establish an accurate diagnosis of PJI, with applicability in sonicated fluid (Šuster and Cör, 2022).

### 6.7 Alternative identification methods

Among the alternative methods of bacterial identification that have been suggested for the diagnosis of infections associated with implants, the BioTimer Assay (BTA), which indirectly identifies microorganisms through the detection of microbial metabolic products, uses an original reagent containing red phenol or resazurin as indicators. Phenol red changes from red to yellow, indicating the presence of fermenting microorganisms, while resazurin changes from violet to pink, indicating the presence of nonfermenting microorganisms (Rosa et al., 2019).

Another method is isothermal microcalorimetry, which is considered a new method for real-time detection of heat production related to the growth of reproductive microorganisms in biological fluid. This detection method has proven to be highly sensitive and rapid in synovial fluid samples for the diagnosis of septic arthritis. Likewise, sonication fluid microcalorimetry was useful for diagnosing PJI with a considerably faster detection time than conventional microbial culture (Borens et al., 2013; Morgenstern et al., 2020).

These methods have the advantages of easy execution and accessibility for the identification process. However, they present important limitations compared to molecular methods, which perform more precise identification. BTA is incapable of identifying microbial genera and species, a problem that can be remedied with sonication, as BTA has good sensitivity for microbial analysis (Rosa et al., 2019).. Microcalorimetry has reduced sensitivity due to the same challenge that culture faces with the presence of biofilms for diagnosing PJI but could complement cultures and support rapid, real-time decisions in orthopedic device-related infections (Morgenstern et al., 2020).

Additionally, imaging techniques to visualize biofilms can also be applied. Confocal laser scanning microscopy and scanning electron microscopy provide imaging of the biofilm without compromising the biofilm structure; in some cases, confocal laser microscopy makes it possible to visualize viable biofilm bacteria in joint fluid, wound tissue and bone cement. Scanning electron microscopy can be used to visualize the coaggregation of microbial cells, but the preparation often results in the loss of the biofilm matrix. The cost and training requirements for obtaining the best images limit the use of these techniques (56).

## 7 Conclusion

One of the primary challenges in the management of implant-associated infections is microbiological diagnosis. To ensure a reliable diagnosis and successful treatment, complete removal of the implant and dislodgment of the microorganisms causing the infection, which are predominantly present in biofilm structures, are necessary. For this purpose, the sonication technique was successfully proposed, although its diagnostic accuracy is still questioned in the current literature.

When reviewing the literature, it was possible to observe the adoption of different protocols, as expected, and consequently different results regarding the sensitivity and specificity of sonicated fluid cultures compared to those of periprosthetic tissue cultures. It was possible to observe an even greater prevalence of coagulase-negative *Staphylococcus* species, followed by *Staphylococcus aureus*, identified as etiological agents of infections associated with implants, by both culture methods, but sonication proved to be important for the identification of low-virulence pathogens that produce biofilms, which are notoriously difficult to detect, such as the species *Peptostreptococcus* and *Corynebacterium* spp.

In the analysis of the studies that compared SFC and chemical methods of biofilm displacement, EDTA and DTT, it was observed that the results varied between the superior sensitivity and specificity of SFC, and there was no significant difference between the SFC and chemical methods.

In this context, we conducted an analysis of various aspects, including sample collection, storage conditions, cultivation methods, microorganism identification techniques (both phenotypic and molecular), and the cutoff point for CFU counts. Additionally, we propose optimal parameters for programming the sonication bath and sample processing.

In conclusion, based on our analysis and review of the current literature, we have established a theoretical foundation for standardizing sonication protocols. The aim of this study was to achieve the highest sensitivity and specificity indices for the reliable microbiological diagnosis of infections associated with implants and prosthetic devices, such as PJIs. However, practical application and further complementary studies are still necessary.

## Author contributions

ND: Data curation, Methodology, Project administration, Writing – original draft, Writing – review & editing. BS: Data curation, Methodology, Writing – original draft, Writing – review & editing. AO: Formal Analysis, Supervision, Validation, Writing – review & editing. PR: Formal Analysis, Resources, Supervision, Validation, Writing – original draft, Writing – review & editing.
